# The London School Board

**Published:** 1897-10-23

**Authors:** 


### The Hospital

A Journal of

The Medical Sciences and Hospital Administration.

Vol. XXIII.—No. 578.] [Oct. 23, 1897.

### The London School Board.

The approaching election of the London School Board has had its customary effect of fanning into renewed activity the embers of politico-theological strife, and of inducing many so-called friends of education to range themselves under the banners of opposing controversialists as a preparation for the impending poll. In the meanwhile, a very shrewd and sensible correspondent of the *Times*, whose identity is veiled under the modest signature of "W.," has endeavoured to remind the electors of the existence of the children. In other words, he has invited consideration of the purposes which schools are intended to fulfil, and of the means by which the fulfilment of these purposes is most likely to be accomplished. If it be the object of the schools to "educate," what ought we to understand by "education?" In its widest sense, as Paley tells us, the word comprises every preparation which is made in our youth for the sequel of our lives; and, somewhat on the same basis, "W." asserts that the first object of education should be to make a child grow up into a good man or woman; the second, to give it a certain amount of useful knowledge to enable it to start and get on in life, not necessarily with an eye to "rising to something higher," but with the habit and purpose of doing its duty manfully, honestly, energetically, and intelligently in whatsoever state of life it may please God to call it to. To inculcate a sense of duty and responsibility should be the first object of education, to impart useful knowledge the second; to teach a great deal of the stuff that is taught in schools nowadays should be no part of it at all.

It would be difficult to read and to consider these vigorous sentences without at least arriving at a suspicion that the modern schoolmaster has greatly lost his way, and that the modern "educationist"—we believe that is the right word—too often furnishes a typical illustration of that blind guide whose chief function is to lead his equally blind neighbour into the ditch. There can be no question but that the power of easily reading printed matter, to take a single instance, has been far more widely diffused abroad during the last five-and-twenty years than was formerly thought possible; but we greatly doubt whether there has been any corresponding increase of intelligence, or whether the newly "educated" classes have become better able to appreciate the weight and meaning of the verbal symbols which they have learnt to recognise by the sense of vision. Hogarth, in one of the best known of his pictures, drew a man seated upon the signboard of an inn, and sawing through its support between himself and the house from which it projected; and, in somewhat similar fashion, the newspapers of to-day present us with a picture of thousands of English workmen who are "on strike," with the utterly futile expectation of being able to obtain higher wages by rendering it impossible for their employers to contend against American and Continental competition. These men who are on strike all read, and probably read every word of the empty promises addressed to them by persons whose interest it is to maintain a state of disorder by which they live; but can it be contended that the reading has been guided to any useful purpose, or that the readers are more accustomed than their fathers to look facts in the face, and to reason upon them for their future guidance? Perhaps the most important part of secular education, for the wage-earning classes, would be to teach them the principles of conduct, the reasons why industry and thrift lead to competence and comfort, the reasons why idleness and extravagance lead to the workhouse; while the most important part of religious education would be the awakening of a sense of duty to the Giver of all good as well as to mankind; but we fear that the records of the School Board do not exhibit any remarkable progress in either of these directions. The average schoolmaster and the average "educationist" seem to us to be living in a fool's paradise, and to be exulting over an appearance of progress which is only now and then relieved by some chance instances of reality. The coming election—if we may take our views of it from the correspondence appearing in the daily papers—is expected to turn in great measure on the question whether or not the Apostles' Creed should form part of the recognised religious teaching. Nothing has been said—as far as we have observed—as to the manner of the teaching, or as to the methods by which the almost archaic sentences of the venerable formulary can be so presented to a child as to awaken any portion of its intelligence, or to give rise to the formation of a single definite idea within its undeveloped brain. That they can be so presented as to produce neither of these results is a fact of which we have abundant evidence. More than forty years ago the Rev. Mr. Brookfield, then an inspector of schools, called upon two children to write down not the Creed, but the answers of the Church Catechism to two questions. The children had been accustomed to repeat these answers every day for four or five years. Their written versions were very much like each other, and here is one of them:—

"My dooty tords my Nabers to love him as thyself and to do to all men as I wed thou shall do and to me to love onner and suke my farther and mother to onner and to bay the queen and all that are pet in a forty under her to smit myself to all my goones teaches sportial pastures and marsters to oughten mysilf lordly and every to all my betters to hut nobody by would nor deed to be trew in jest in all my deelins to beer no malis nor ated in your arts to kep my ands from pecken and steel my turn from evil speak and lawing and slanders not to civet nor desar othermans good but to lern laber trewly to git my own leaving and to do my dooty in that state if life and to each it his please to god to call men."

Is it likely that the Creed, taught in the most approved School Board fashion, will have any better result, either moral or intellectual, than is indicated by the foregoing?

